# A Positive Newborn Screen for Congenital Hypothyroidism in a Clinically Euthyroid Neonate—Avoiding Unnecessary Treatment

**DOI:** 10.3390/ijns9020016

**Published:** 2023-03-23

**Authors:** Ashleigh Brown, Paul Hofman, Bobby Li, Campbell Heron, Natasha Heather

**Affiliations:** 1 Starship Child Health, Te Whatu Ora Te Toka Tumai Auckland, Auckland 1023, New Zealand; 2 Liggins Institute, University of Auckland, Auckland 1023, New Zealand; 3 Canterbury Health Laboratories, Te Whatu Ora Waitaha Canterbury, Christchurch 8011, New Zealand; 4 LabPlus, Te Whatu Ora Te Toka Tumai Auckland, Auckland 1148, New Zealand

**Keywords:** newborn screening, congenital hypothyroidism, familial dysalbuminaemic hyperthyroxinaemia, albumin variant, euthyroid, discordant

## Abstract

Newborn screening for congenital hypothyroidism (CH) has dramatically improved the neurocognitive outcomes for newborns with a confirmed positive screening test result. However, screening yields a small number of false positive and false negative results. This report describes the first known case of familial dysalbuminaemic hyperthyroxinaemia presenting with a positive newborn thyroid stimulating hormone screen. This condition is characterized by artefactually elevated free tetraiodothyronine (T4) and triiodothyronine (T3) levels due to increased albumin binding and subsequent dissociation during laboratory assays but normal true free thyroid hormone and thyroid stimulating hormone (TSH) levels in a clinically euthyroid subject. This highlights the need to take care when attributing clinical significance to discordant results.

## 1. Introduction

Newborn screening for congenital hypothyroidism has dramatically improved the neurocognitive outcomes for newborns with a confirmed positive screening test result. However, screening yields a small number of false positive and false negative results. This report describes the first known case of familial dysalbuminaemic hyperthyroxinaemia presenting with a positive newborn screen and highlights the need to take care when attributing clinical significance to discordant results.

T4 and T3 are hydrophobic and require plasma transport proteins to get to target organs. The majority of T4 and T3 hormones are bound to three thyroid hormone (TH) transport proteins: thyroxin-binding globulin (TBG), transthyretin (TTR) and human serum albumin (albumin). Most TH binding is to TBG (75%), followed by TTR (15–20%), with albumin binding the least (5% of T4 and 20% of T3). Albumin is the most abundant protein in circulation and has more binding sites for TH than the others, but it has the lowest binding affinity [1,2].

Familial dysalbuminaemic hyperthyroxinaemia (FDH) is caused by a missense mutation in the albumin (ALB) gene on chromosome 4, which causes a subtle change in the structure of albumin and increases its binding affinity for T4 or T3 [3,4]. Thus, there is increased total T4/T3 but normal free T4, free T3, and TSH levels. However, commercial T4 and T3 assays frequently report artefactually elevated free T4 and/or free T3 levels in this condition. This report describes an infant with this mutation, which we speculate is the cause of a false positive newborn screening for TSH elevation.

## 2. Case Report

Baby A, a South Korean female, was referred for evaluation of possible congenital hypothyroidism at 4 days of age with elevated TSH on a newborn metabolic screening. Baby A was born at 37 + 5 weeks gestation via normal vaginal delivery. The pregnancy was unremarkable other than a maternal diagnosis of subclinical hypothyroidism with a serum TSH of 2.7 mU/L in the first trimester, for which the mother received 25 micrograms of thyroxine daily throughout the pregnancy (Roche assay, reference interval 0.27–4.2 mU/L, local recommendation for serum TSH < 2.5 mU/L in pregnancy). Thyroid antibody testing was not performed. Baby A’s elder brother had a positive newborn screen for congenital hypothyroidism in South Korea but was never treated due to normal follow-up TFTs.

Baby A’s newborn screening TSH level was 22 mU/L in whole blood on a dried blood spot sample collected at 32 h of age. This met New Zealand criteria for a positive screen and direct referral for diagnostic assessment (TSH ≥ 20 mU/L whole blood, GSP, Perkin Elmer) [5]. A blood sample was collected for diagnostic serum TFTs, and she was prescribed a loading dose of L-thyroxine at 10 mcg/kg/day, to be taken after review of TFTs [6]. Serum TFTs demonstrated high free T4 of 54 pmol/L (10–40) and a mildly elevated TSH of 14.8 mU/L. Her family was advised not to commence thyroxine, as the results were inconsistent with hypothyroidism. Repeat TFTs were similar 3 days later. Baby A was clinically euthyroid, thriving, gaining weight, and waking as expected for feeds. Ongoing monitoring of thyroid function continued to show discordant results, with an elevated T4 and non-suppressed TSH (see Table 1).

Due to the discordant TFTs, the following additional tests were performed to rule out interference in the biochemistry analyser and other artefacts: precipitation of antibodies and other large molecules/complexes with polyethylene glycol, treatment with heterophile antibody-blocking reagent, and serial dilution with normal human serum. In all cases, there was no gain or loss of recovery of TSH or free T4, suggesting that the unusual TFTs were not explained by the presence of macro-TSH, the presence of antibodies, or other factors that interfere with the analytical process. The free T4 analysed by Siemens Atellica was near the upper reference limit, while the total T4 on a Beckman Coulter immunoassay on day 15 was significantly higher than the upper reference limit (Table 1).

**Figure 1:**
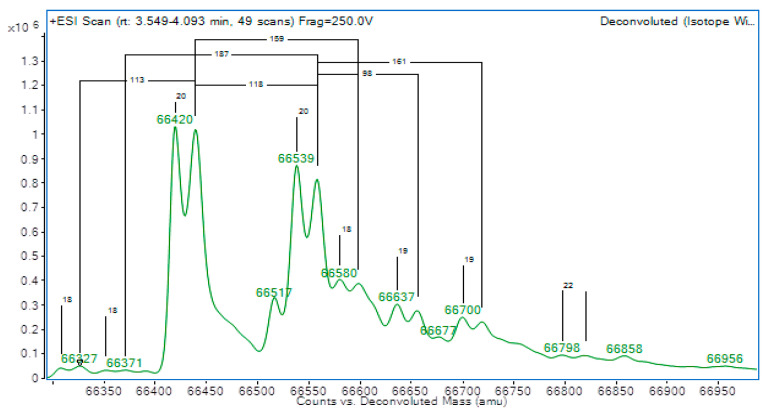
Albumin mass spectrometry showing two double peaks at 66,440 Da & 66,559 Da, indicating heterozygosity for a normal and variant albumin.

The initial differential diagnoses included thyroid hormone resistance and a thyroid hormone transport protein variant. Albumin analysis was then performed by liquid chromatography time-of-flight mass spectrometry (LC-TOF-MS) to investigate possible TH-binding variants. Apart from expected albumin (66,440 Da) and cysteinylated albumin peaks (66,559 Da), there were also clear peaks approximately 19 Da below the wild-type albumin peaks (66,420 and 66,539 Da), suggesting heterozygosity for a normal and a variant albumin (see Figure 1).

Baby A’s genetic testing confirmed the diagnosis of FDH with heterozygosity of the NM_000477.5 (ALB):c.725G > A (p.Arg242His) variant in the albumin gene, or Arg218His in protein nomenclature, thus confirming the diagnosis of FDH.

## 3. Discussion

This report describes a well-recognised abnormality in albumin causing abnormal protein binding of T4 that has never been considered before in the interpretation of discordant follow-up test results of a newborn screen for CH. The elevated newborn screening dried blood spot TSH level suggests that this abnormality led to a transient, true reduction in free T4, which rapidly normalised postnatally. This is a previously unrecognised cause of a positive newborn TSH screening test. This case was puzzling, as while in-vivo free T4 levels are normal, as evidenced by a rapidly normalized TSH level, many commercial free T4 assays will produce artefactually elevated free T4 results in this condition.

FDH is an autosomal dominant condition affecting the binding affinity of thyroid hormones to albumin. This results in elevated total T3 or T4 levels but normal free T3, free T4, and TSH levels [2]. It is the most common inherited cause of artefactually high serum free T4 levels in euthyroid subjects [7]. There are a number of described mutations, with the most common being that observed in Baby A (Arg218His), where arginine is substituted for histidine at position 218 on the albumin protein [4]. This variant has previously been described in Korean patients, but only since 2016 [5]. There are two types of FDH, FDH-T3 and FDH-T4, resulting in higher affinity binding for their respective hormones [1]. The prevalence varies by ethnicity, as common as 1 in 50 in some Hispanic populations (particularly Puerto Rican) and up to 1 in 1000 in non-Hispanic white populations [1]. As the condition is inherited in a dominant manner, family history may reveal relatives receiving inappropriate treatment and enable screening of siblings and offspring to prevent unnecessary concern and investigation in the future.

This condition can be difficult to detect because despite the free and biologically active TH levels being normal, the testing process causes dissociation of TH from albumin. This results in the measurement of both free TH and a fraction of the bound TH, resulting in artefactual elevation of free T4 [2]. Dissociation is a known phenomenon in assays but is usually small in scale, predictable and offset by calibration [8]. The effect is somewhat assay dependent, and variation exists even within the same assay, as shown in Table 1.

Most clinical laboratories use indirect free TH quantitation methods, as they are faster, simpler, and less expensive than direct methods, such as equilibrium dialysis or ultracentrifugation. Indirect quantitation of free TH assumes the given fraction of measured free TH has come from the bound fraction and adjusts accordingly. This works well for the vast majority of patients. However, in FDH, a much larger quantity of free TH is bound to albumin and dissociates during analysis, producing spurious increased results. If a direct method were available in this case, normal free T4 results would be expected.

Additionally, albumin can be analysed by liquid chromatography time-of-flight mass spectrometry (LC-TOF-MS) to clarify the cause of the discordant results. The masses of intact albumin can be assessed and compared to either a normal albumin or a patient with FDH. In the context of discordant TFTs, based on literature and local experience, a mass change of approximately −19 Da is highly suggestive of FDH due to Arg218His, whereas the presence of normal masses alone is consistent with normal albumin [9]. As an alternative screening approach, iso-electric focusing can also be used [10]. Although peptide mapping can then be used to narrow down the location of the observed mass change and the exact amino acid involved, in practice, it is simpler to perform ALB exon 7 sequencing to confirm the variant if required, as was performed in this case.

The limitations of this case report include the fact that only partial TFT results were available in family members. FDH is inherited in an autosomal dominant manner, and we suspect that the sibling’s positive newborn screen may also have been caused by FDH. However, he was born overseas, and the details of his newborn screen and diagnostic TFTs were not accessible to us. The presence of maternal thyroid antibodies is an alternative explanation for a transient neonatal increase in TSH, although this invokes a second unproven pathology. Furthermore, reference intervals for TSH levels during pregnancy are unclear and assay dependent [11], and we acknowledge that the mother’s first trimester TSH level was not definitively elevated.

## 4. Conclusions

This case highlights the need for caution in interpreting positive newborn screening results and discordant thyroid function tests, particularly when results don’t fit a typical pattern and the associated clinical picture. FDH is most easily diagnosed by genetic testing for the albumin variant or alternatively can be assayed by liquid chromatography time-of-flight mass spectrometry (LC-TOF-MS) where available.

## Figures and Tables

**Table 1:** Serial TFTs (serum) by different assays across four laboratories.

		Roche Assay			Siemens Atellica Assay			Beckman Coulter Assay	
		TSH (mU/L)	Free T4 (pmol/L)	Free T3 (pmol/L)	TSH (mU/L)	Free T4 (pmol/L)	Free T3 (pmol/L)	Total T4 (nmol/L)	Total T3 (nmol/L)
	Age-Specific Reference Interval	0.4–16.0	10–40	3–10	1.0–8.0	15–35	3–10	76–168	1.4–3.96
Day 4	Lab ‘B’	14.8	54						
Day 5	Lab ‘A’	7.2	52.5						
Day 10	Lab ‘A’	7.2	42.8	7.3					
	Lab ‘B’	8.1	47	7.2					
	Lab ‘C’				5.7	34	9.5		
Day 15	Lab ‘B’	8.3	37	7.1					
	Lab ‘D’							299	2.1
Day 29	Lab ‘A’	5	29.6	7					

## Data Availability

All data is contained within the article.

## References

[1] Mimoto M.S., Refetoff S. (2020). Clinical recognition and evaluation of patients with inherited serum thyroid hormone-binding protein mutations. J. Endocrinol. Investig..

[2] Khoo S., Lyons G., McGowan A., Gurnell M., Oddy S., Visser W.E., Berg S.V.D., Halsall D., Taylor K., Chatterjee K. (2020). Familial dysalbuminaemic hyperthyroxinaemia interferes with current free thyroid hormone immunoassay methods. Eur. J. Endocrinol..

[3] Kragh-Hansen U., Galliano M., Minchiotti L. (2017). Clinical, Genetic, and Protein Structural Aspects of Familial Dysalbuminemic Hyperthyroxinemia and Hypertriiodothyroninemia. Front. Endocrinol..

[4] Pappa T., Ferrara A.M., Refetoff S. (2015). Inherited defects of thyroxine-binding proteins. Best Pr. Res. Clin. Endocrinol. Metab..

[5] Huynh T., Greaves R., Mawad N., Greed L., Wotton T., Wiley V., Ranieri E., Rankin W., Ungerer J., Price R. (2022). Fifty years of newborn screening for congenital hypothyroidism: Current status in Australasia and the case for harmonisation. Clin. Chem. Lab. Med..

[6] Heather N., Hofman P. (2021). Congenital Hypothyroidism—Early Assessment and Management. Starship Child Health Clinical Guidelines. https://starship.org.nz/guidelines/congenital-hypothyroidism-early-assessment-and-management/.

[7] Cho Y.Y., Song J.-S., Park H.-D., Kim Y.N., Kim H.-I., Kim T.H., Chung J.H., Ki C.-S., Kim S.W. (2017). First Report of Familial Dysalbuminemic Hyperthyroxinemia With an ALB Variant. Ann. Lab. Med..

[8] Freedman D.B., Halsall D., Marshall W.J., Ellervik C., Rifai N.H. (2018). Thyroid Disorders. Tietz Textbook of Clinical Chemistry and Molecular Diagnostics.

[9] Ryan J.B., Brennan S.O., Potter H., Wolmarans L., Florkowski C.M., George P.M. (2016). Familial dysalbuminaemic hyperthyroxinaemia: A rapid and novel mass spectrometry approach to diagnosis. Ann. Clin. Biochem..

[10] Flechner I., Aranoff G., Reifen R., Landau H. (1992). Detection of albumin binding abnormalities in sera of patients with familial dysalbuminaemic hyperthyroxinaemia using isoelectric focusing. Endocr. Res..

[11] Okosieme O.E., Agrawal M., Usman D., Evans C. (2021). Method-dependent variation in TSH and FT4 reference intervals in pregnancy: A systematic review. Ann. Clin. Biochem. Int. J. Biochem. Lab. Med..

